# The causal link between hyperchloremia and acute kidney injury is yet to be conclusively established: we are not sure

**DOI:** 10.1186/s13054-020-02966-8

**Published:** 2020-05-28

**Authors:** Patrick M. Honore, Aude Mugisha, Luc Kugener, Sebastien Redant, Rachid Attou, Andrea Gallerani, David De Bels

**Affiliations:** ICU Department, Centre Hospitalier Universitaire Brugmann-Brugmann University Hospital, Place Van Gehuchtenplein, 4, 1020 Brussels, Belgium

We read with interest the recent article by Williams et al. comparing Plasma-Lyte-A with 0.9% saline in children with diabetic ketoacidosis (DKA) [1]. They conclude that both fluids were similar in regard to time to resolution of DKA, need for renal replacement therapy (RRT), mortality, and lengths of pediatric intensive care unit (PICU) and hospital stay [1]. In addition, the incidence of new or progressive acute kidney injury (AKI) and resolution of AKI were similar in both groups. They conclude that the cause for hyperchloremia seems more of a normal physiological response to loss of bicarbonate over chloride with improved renal perfusion than due to fluid type or volume. We would like to make some comments. The chloride elevation in this study was very modest in both groups—only 4 mmol/L for the Plasma-Lyte group and 6 mmol/L for the normal saline group [1]. If we look at the study of Baalaaji et al. [2], where all the patients were resuscitated with saline, the chloride in the non-AKI group was 106.6 mmol/L versus 118.6 mmol/L in the AKI group, a difference in chloride of 12 mmol/L [2]. In that study, twenty-eight (35%) children were diagnosed with AKI: twenty (71.4%) recovered with hydration alone while 8 (28.6%) required RRT [2]. None of the admission variables could predict AKI; however, on multivariable analysis, elevated chloride levels at 24 h had an independent association with AKI progression [adjusted OR 1.14 (95% CI 1.04–1.27), *P* = 0.007]. Serum chloride > 112 mmol/L at 24 h had a sensitivity, specificity, and area under ROC curve of 73.3%, 82.4%, and 0.835, respectively, for development of AKI (*P* < 0.001) [2]. Although children with “AKI progression” had higher PRISM III and admission chloride levels, only the 24-h serum chloride was independently associated with “AKI progression.” Independent association of the 24-h serum chloride rather than admission value leads one to believe that hyperchloremia could have been an iatrogenic element caused by the type of intravenous fluids received [2]. Hyperchloremia has been hypothesized to cause renal hypoperfusion and AKI by virtue of its renal vascular smooth muscle constrictor effect [3, 4]. A causal link between hyperchloremia and AKI has yet to be proven *or disproven*. Further randomized controlled studies are needed to elucidate the relationship between fluids and AKI.

## Authors’ response

### Hyperchloremia and acute kidney injury: chicken or egg?

Vijai Williams, Muralidharan Jayashree

Authors response to the letter

We thank Honore and colleagues for their interest in our recent work [1]. The link between hyperchloremia and acute kidney injury (AKI) has been a topic of intense debate both in septic shock and DKA [5, 6]. However, as the authors have rightly pointed out, a causal link between both is yet to be proven. More importantly, it is a moot point whether hyperchloremia is a cause or effect of AKI.

In this context, the study by our group (Baalaaji et al.) [2] inferred that AKI followed hyperchloremia. The difference in chloride levels from admission to 24 h was higher in children with progressive AKI as compared to the no AKI group [118.6 mmol/L vs. 106.6 mmol/L]. This was attributed to prolonged use of 0.9% saline, due to scarce availability of 0.45% saline. We had concluded that hyperchloremia possibly resulted in higher incidence of AKI and need for RRT. But it was a retrospective analysis with inherent limitations, wherein the type of fluids, their volume, and duration were not quantified. That is precisely the reason why we planned the current randomized controlled trial. We wanted to ascertain the causal link between hyperchloremia and AKI as observed in our previous study.

The current study revealed different results. Firstly, admission chloride levels were higher in both arms [112.5 (103.7 to 117.7) mmol/L in Plasma-Lyte-A and 111 (105.2 to 117.7) mmol/L in 0.9% saline group]. The possible reasons could be higher incidence of baseline AKI and/or administration of fluids with higher chloride content prior to referral. Since the latter was an exclusion criterion for the trial, we could infer that hyperchloremia is a consequence of AKI. Secondly, the difference in chloride elevation with time in saline and Plasma-Lyte was marginal and similar. The higher baseline chloride levels possibly minimized this difference in delta chloride levels. Thirdly, the need for RRT as compared to our previous study was low (3% vs. 28%) arguing against hyperchloremia as a cause for AKI.

Putting all together, it seems that most AKI in DKA resolves with adequate hydration, irrespective of the type of fluids used. Whether the higher baseline chloride levels before hydration are a manifestation of impaired renal perfusion is conjectural. As we seek more answers, the link between hyperchloremia and AKI is akin to the proverbial chicken and egg.

## Data Availability

Not applicable.

## Abbreviations

AKI: Acute kidney injury
DKA: Diabetic ketoacidosis
RRT: Renal replacement therapy
PICU: Pediatric intensive care unit
